# Supplementary figures and images for: Source attribution of community-acquired cases of Legionnaires’ disease–results from the German LeTriWa study; Berlin, 2016–2019
Source: PLoS One. 2020 Nov 25;15(11):e0241724. doi: 10.1371/journal.pone.0241724 (PMC7688155; doi:10.1371/journal.pone.0241724)

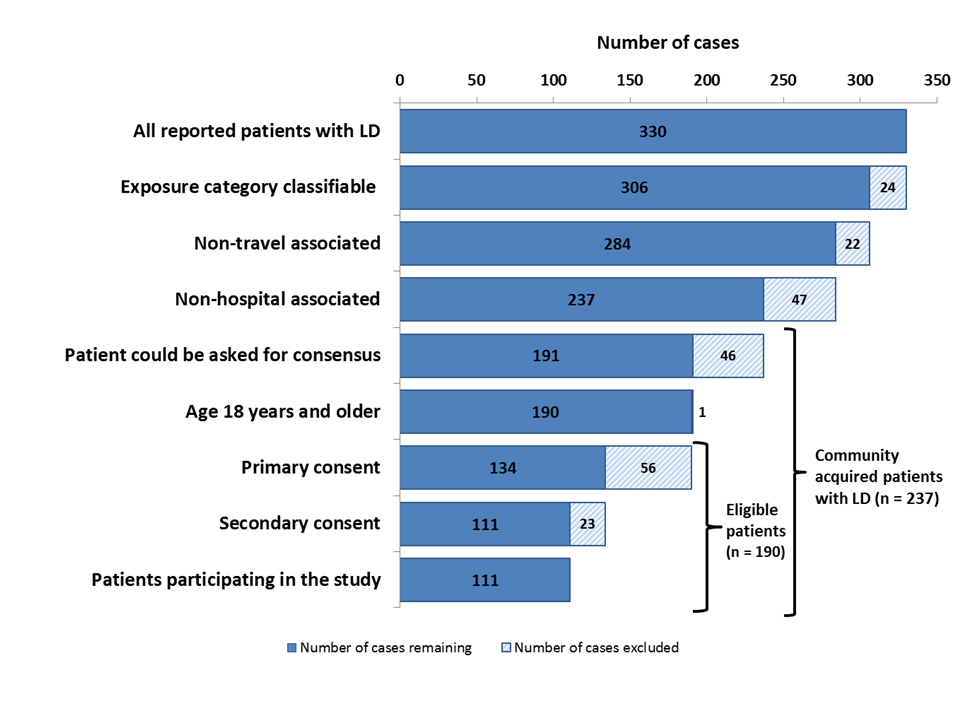

Supplement: S1 Fig — LD = Legionnaires’ disease. In red: number of patients where the respective category applies; Berlin, 2016–2019. (TIF) [file pone.0241724.s001.tif]

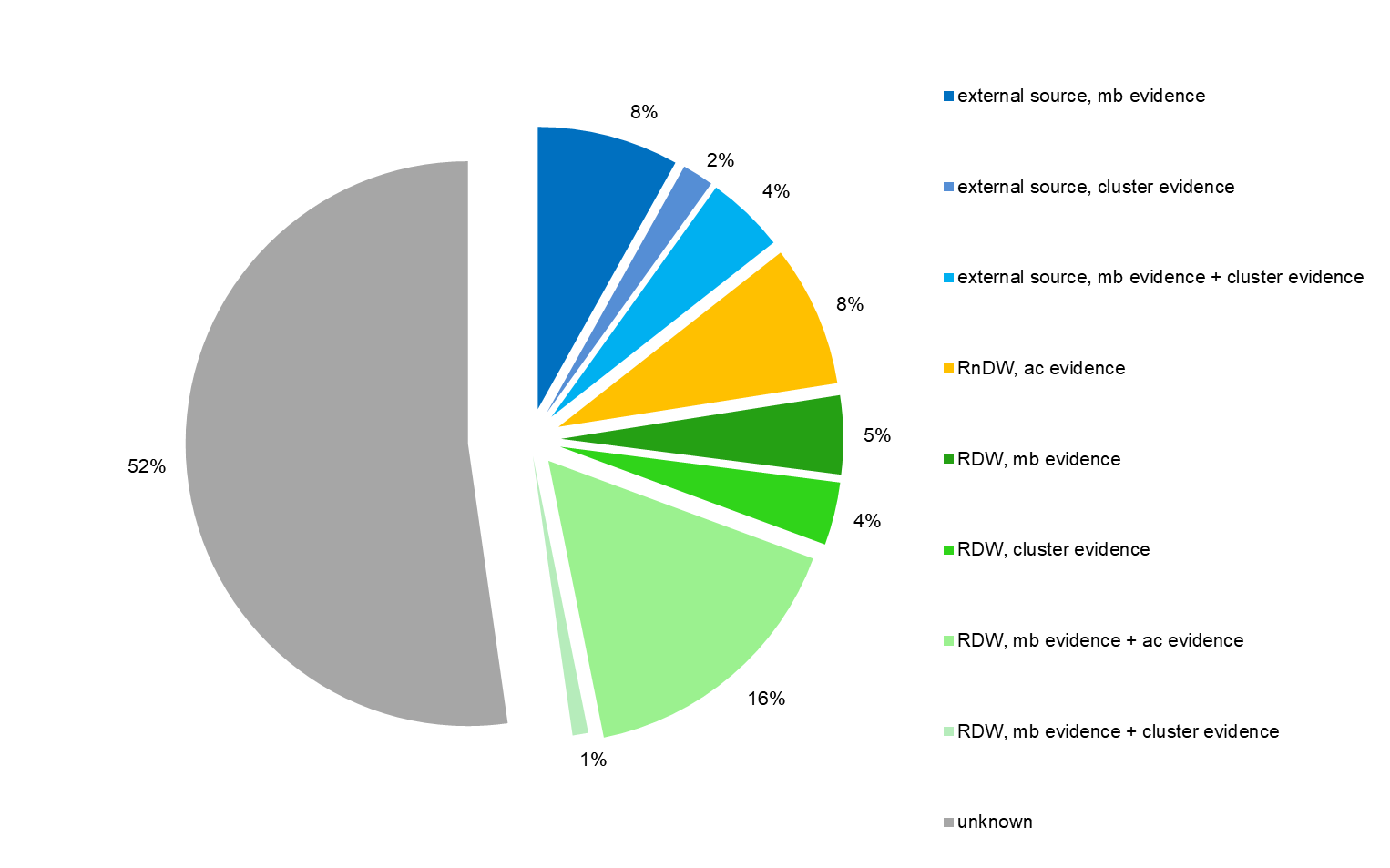

Supplement: S2 Fig — (TIF) [file pone.0241724.s002.tif]
