# Supplementary material for: Source attribution of community-acquired cases of Legionnaires’ disease–results from the German LeTriWa study; Berlin, 2016–2019
Source: PLoS One. 2020 Nov 25;15(11):e0241724. doi: 10.1371/journal.pone.0241724 (PMC7688155; doi:10.1371/journal.pone.0241724)
Supplement: S1 Material — (DOCX) [file pone.0241724.s003.docx]

**S1 Material.**

Questions asked in the case – control questionnaire

# Individual risk factors

- Age
- Sex
- educational level (highest degree)
- profession
- daily typical activities
- need for assistance with daily activities, e.g. in personal hygiene
- living in one or two households
- duration of absence at residence (in days)
- type of building of residence
- number of floors of building of residence
- number of floors of the residential building
- medical preconditions / underlying illnesses
- smoking status
- alcoholic cosumption
- having heard of Legionnaires‘ disease before illness onset
- practices to prevent acquisition of Legionnaires‘ disease before illness onset
- household size
- age, sex of household contacts
- history of Legionnaires‘ disease in household in the previous six months
- history of acute respiratory illnesses in household in the previous six months

# Health related quality of life 4 weeks before and half a year after disease onset (according to SF-12 Health Survey (www.hogrefe.de/shop/fragebogen-zum-gesundheitszustand.html))

- General Health
- Physical Functioning
- Role Functioning (Physical)
- Bodily Pain
- Vitality
- Role Functioning (Emotional)
- Mental Health
- Social Functioning

# External sources or factors pointing towards an external source

- potential exposure to water / aerosol during period of infection…
  - when having a shower outside of one‘s own residence
  - when visiting a swimming pool
  - in the sauna/wellness facility
  - at a lake used for swimming
  - in a whirlpool
  - in a car wash
  - when using or standing nearby a high pressure device
  - near a water fountain
  - near a mist machine for plants or fruits/vegetables
  - near a lawn watering machine / sprinkler
  - in a commercial garden center
  - decorative indoor fountains, indoor waterfalls or other artificial water installations
  - when handling or standing nearby industrially used water, e.g. for cooling
  - near an asphalt paving machine
  - when staying nearby a cooling tower / evaporative condenser
  - near wastewater
  - earthworks, excavation, digging near the place of residence
  - distance to the nearest large road with heavy traffic (as defined by the patient)
  - use of public transport
  - riding a car (riding oneself or being taken by s.o. else)
  - riding a lawry/truck (riding oneself or having been taken by s.o. else)
    - if yes: was the windscreen wiper used?
    - what was the compostion of the liquid for the windscreen washer system (commercial product / drinking water mixed with cleaning solution / pure drinking water without additives)
  - professional car driving
  - riding a car or having been taken by someone in a car when streets were wet because of rain
  - going for a walk when streets were wet because of rain
  - use of a mobile home

# Residential non-drinking water sources or factors pointing towards a residential non-drinking water source

- potential exposure to water / aerosol during period of infection…
  - using a water filter
  - using a device to produce fizzy gas in drinking water
  - using a continuous positive airway pressure mask
  - using a fish tank
  - using a room humidifier
  - using an indoor fountain
  - using a device for inhalational therapy
  - upon receiving physical treatment using water
  - upon receiving a cosmetic treatment using water mist
  - when having gone to the hairdresser
  - when having gone to the dentist
  - during the birth of a child
  - during work in the garden
  - when using a garden hose
  - when using a garden shower
  - use of denture (yes/no; at daytime; at night time)
    - number of days of use of denture during period of infection
    - method of denture cleaning
    - cleaning of denture only with clear drinking water
    - cleaning of denture only with clear drinking water and tooth paste
    - cleaning of denture only with desinfectant solution
    - keeping denture in container with desinfectant solution
      - if yes, how often was desinfectant solution changed
      - how long was denture soaking in desinfectant solution
    - duration of placement of denture in container with desinfectant solution
    - placement of denture in container with only drinking water
      - if yes: how often was the water changed
      - how long was denture soaking in water
  - cleaning of teeth with dental floss or professionally at dentist
  - cleaning of mouth before going to sleep with desinfectant mouth solution
  - handling of earth
  - handling of compost

# Residential drinking water sources or factors pointing towards residential drinking water

- type of piped water system of the residential building
- preset temperature of warm water
- presence of floor heating in the bathroom
- use of an additional, non-communal water source, such as private well
- use of shower
- use of bath tub
  - approximate temperature of water used in bath tub
- drinking of water directly from the tap (warm/luke warm/cold water)
- flushing of water before use of water at home
- number of days during which the residence was not inhabited during period of infection
  - use of shower/bath tub after the apartment/house was not inhabited
- showering habits of household members (who first, how long)
- bathing habits of household members (who first, how long)
- presence in bathroom during filling of bath tub with water for bathing
- number of times cleaning one’s teeth
- brushing teeth in the morning / during the day / in the evening
- brushing teeth under the shower
- potential exposure to water / aerosol during period of infection…
  - washing the dishes
  - using a dishwasher
  - using a washing machine
  - performing a mouth wash
  - using an electric tooth brush
- problems with / history of repair of piped water installation (during period of infection)
- history of repairing the communal water supply near the place of residence
- use of ice cubes
