# Supplementary material for: Source attribution of community-acquired cases of Legionnaires’ disease–results from the German LeTriWa study; Berlin, 2016–2019
Source: PLoS One. 2020 Nov 25;15(11):e0241724. doi: 10.1371/journal.pone.0241724 (PMC7688155; doi:10.1371/journal.pone.0241724)
Supplement: S2 Material — The “best” water sample is the one taken from a source with Legionella strains most likely having caused the infection. (DOCX) [file pone.0241724.s004.docx]

**S2 Material.**

Microbiological results of all patients as well as the result of the “best” water sample taken. The “best” water sample is the one taken from a source with Legionella strains most likely having caused the infection.
